# Cholecystokinin B receptor agonists alleviates anterograde amnesia in cholecystokinin-deficient and aged Alzheimer's disease mice

**DOI:** 10.1186/s13195-024-01472-1

**Published:** 2024-05-15

**Authors:** Nan Zhang, Yixuan Sui, Peter Jendrichovsky, Hemin Feng, Heng Shi, Xu Zhang, Shenghui Xu, Wenjian Sun, Huatang Zhang, Xi Chen, Micky D. Tortorella, Jufang He

**Affiliations:** 1 Department of Neuroscience and Biomedical Sciences, City University of Hong Kong, Hong Kong SAR, 0000 P.R. China; 2 Centre for Regenerative Medicine and Health, Hong Kong Institute of Science & Innovation, Chinese Academy of Sciences, Hong Kong SAR, 0000 P.R. China; 3 Department of Biomedical Engineering, Johns Hopkins University, Baltimore, MD 21205 USA; 4 Department of Neurosurgery, Stanford University, Stanford, CA 94305 USA; 5 National Engineering Laboratory of Big Data System Computing Technology, Shenzhen University, Shenzhen, 518507 P.R. China; 6 Laboratory Testing Division, WuXi AppTec (Suzhou) Co., Ltd, Suzhou, 215104 P.R. China; 7 Zilkha Neurogenetic Institute, University of Southern California, Los Angeles, CA 90033 USA; 8 Guangzhou Institutes of Biomedicine and Health, Chinese Academy of Sciences, Guangzhou, 510530 P.R. China

**Keywords:** Cholecystokinin, Cholecystokinin B receptor agonist, Anterograde amnesia, Alzheimer’s disease, HT-267

## Abstract

**Background:**

As one major symptom of Alzheimer’s disease (AD), anterograde amnesia describes patients with an inability in new memory formation. The crucial role of the entorhinal cortex in forming new memories has been well established, and the neuropeptide cholecystokinin (CCK) is reported to be released from the entorhinal cortex to enable neocortical associated memory and long-term potentiation. Though several studies reveal that the entorhinal cortex and CCK are related to AD, it is less well studied. It is unclear whether CCK is a good biomarker or further a great drug candidate for AD.

**Methods:**

mRNA expressions of CCK and CCK-B receptor (CCKBR) were examined in two mouse models, 3xTg AD and CCK knock-out (CCK^−/−^) mice. Animals’ cognition was investigated with Morris water maze, novel object recognition test and neuroplasticity with *in-vitro* electrophysiological recording. Drugs were given intraperitoneally to animals to investigate the rescue effects on cognitive deficits, or applied to brain slices directly to explore the influence in inducement of long-term potentiation.

**Results:**

Aged 3xTg AD mice exhibited reduced CCK mRNA expression in the entorhinal cortex but reduced CCKBR expression in the neocortex and hippocampus, and impaired cognition and neuroplasticity comparable with CCK^−/−^ mice. Importantly, the animals displayed improved performance and enhanced long-term potentiation after the treatment of CCKBR agonists.

**Conclusions:**

Here we provide more evidence to support the role of CCK in learning and memory and its potential to treat AD. We elaborated on the rescue effect of a promising novel drug, HT-267, on aged 3xTg AD mice. Although the physiological etiology of CCK in AD still needs to be further investigated, this study sheds light on a potential pharmaceutical candidate for AD and dementia.

**Supplementary Information:**

The online version contains supplementary material available at 10.1186/s13195-024-01472-1.

## Background

Currently the dementia population worldwide has exceeded 55 million, and Alzheimer's disease (AD) as well as other forms of dementia have leaped into the top ten causes of death (https://www.who.int). AD is a neurodegenerative disorder and AD patients' brains contain many amorphous aggregates of amyloid β-peptide (Aβ) and intracellular fibrillar aggregates of the microtubule-associated protein tau [1–3]. Plaques and tangles are present mainly in brain regions involved in learning and memory, such as the entorhinal cortex (EC), hippocampus, basal forebrain and amygdala [4, 5]. The EC is among the earliest neuroanatomical structures pathologically affected in AD, showing the highest levels of Aβ-42 and brain atrophy [6–8]. Episodic memory impairment in patients with AD is correlated with EC atrophy [9]. For mild AD patients, EC was the first region that showed significant neuron death [10] and the early affected brain region that can be observed in the brain imaging of AD patients [8]. Early astrocytic atrophy appears in the EC of a triple transgenic mouse model (3xTg mouse) for AD [11].

Removal of the bilateral temporal lobe (including the hippocampus and the EC) prevents the formation of declarative memory in the patient HM, exhibiting the symptom of anterograde amnesia [12–14]. The EC is strongly and reciprocally connected with both the neocortex and hippocampus [15, 16]. We infer that the EC sends a “plasticity-enabled” signal to the neocortex to switch on the encoding of new associative memory through the entorhinal-neocortical projections [17]. Consistent with the previous reports of heavy cholecystokinin (CCK) labelling in the entorhinal and perirhinal cortices [18–20], we further indicated that neurons in the EC projecting to the auditory cortex are mostly CCK immunopositive [17]. CCK, the most abundant neuropeptide [21], is mainly mediated by CCK B receptor (CCKBR) in the brain [22]. The entorhinal CCK enables neuroplasticity and associative memories between two auditory stimuli and between visual and auditory stimuli in the auditory cortex and between auditory and fear stimuli in the amygdala [17, 23–26]. N-methyl-D-aspartate receptors on the cortical projection terminals of the entorhinal glutamatergic CCK neurons trigger the release of CCK after high-frequency stimulation (HFS) [24]. CCKBR mediates the CCK signalling to induce long-term potentiation (LTP) in the neocortex [24].

Decreased CCK mRNA expression is closely correlated with the progression of aging at the AD vulnerable regions (e.g. EC layer 2), indicating qualifying CCK as a biomarker of the disease [27]. A recent study reports that higher CCK levels in the cerebrospinal fluid correlate with better memory scores and a decreased likelihood of AD impairment [28]. We assume that CCK, released in the neocortex by CCK positive neurons in the EC, which is the earliest site of atrophy in dementia patients, is the key chemical for memory encoding, and reduced CCK leading to a deficiency in encoding recent memory or anterograde amnesia in AD patients. We hypothesize that CCKBR agonists are good candidates to treat the anterograde amnesia. We adopted Morris water maze (MWM) and novel object recognition (NOR) tests to examine the above hypothesis on CCK deficient (CCK knockout, short for CCK^−/−^) and 3xTg AD mice and utilized the brain-slice assay to correlate the LTP assay in the hippocampus with their behavioural deficiencies. We examined a natural CCKBR agonist (CCK tetrapeptide, CCK-4) and a self-synthesized CCKBR agonist, named as HT-267, in rescuing behavioural and LTP deficits of both CCK^−/−^ and 3xTg mice .

## Methods

### Animals

All experimental procedures were conducted in accordance with the Animal Subjects Ethics Sub-Committees of the City University of Hong Kong. C57 (C57/BL/6) were obtained from Laboratory Animal Research Unit (LARU, City University of Hong Kong). CCK-knockout [*Cck*^*tm2.1(Cre/ERT2)Zjh*^ /J, stock #012710, CCK^−/−^ for short] were obtained from the Jackson Laboratory. 3xTg AD [B6;129-Tg(APPSwe, tauP301L)1Lfa *Psen1*^*tm1Mpm*^ /Mmjax, stock #034830-JAX] and control wildtype 129 (129S1/SvImJ, stock #002448) mice were obtained from the Jackson Laboratory. Mice were housed in 12 h light / 12 h dark cycle (light on from 8:00 to 20:00) and given food and water ad libitum. However, a week before the behavioral tests, animals were moved to a room with reversed 12 h dark / 12 h light cycle (light off from 8:00 to 20:00).

### Morris Water Maze (MWM) test

**Fig. 1:**
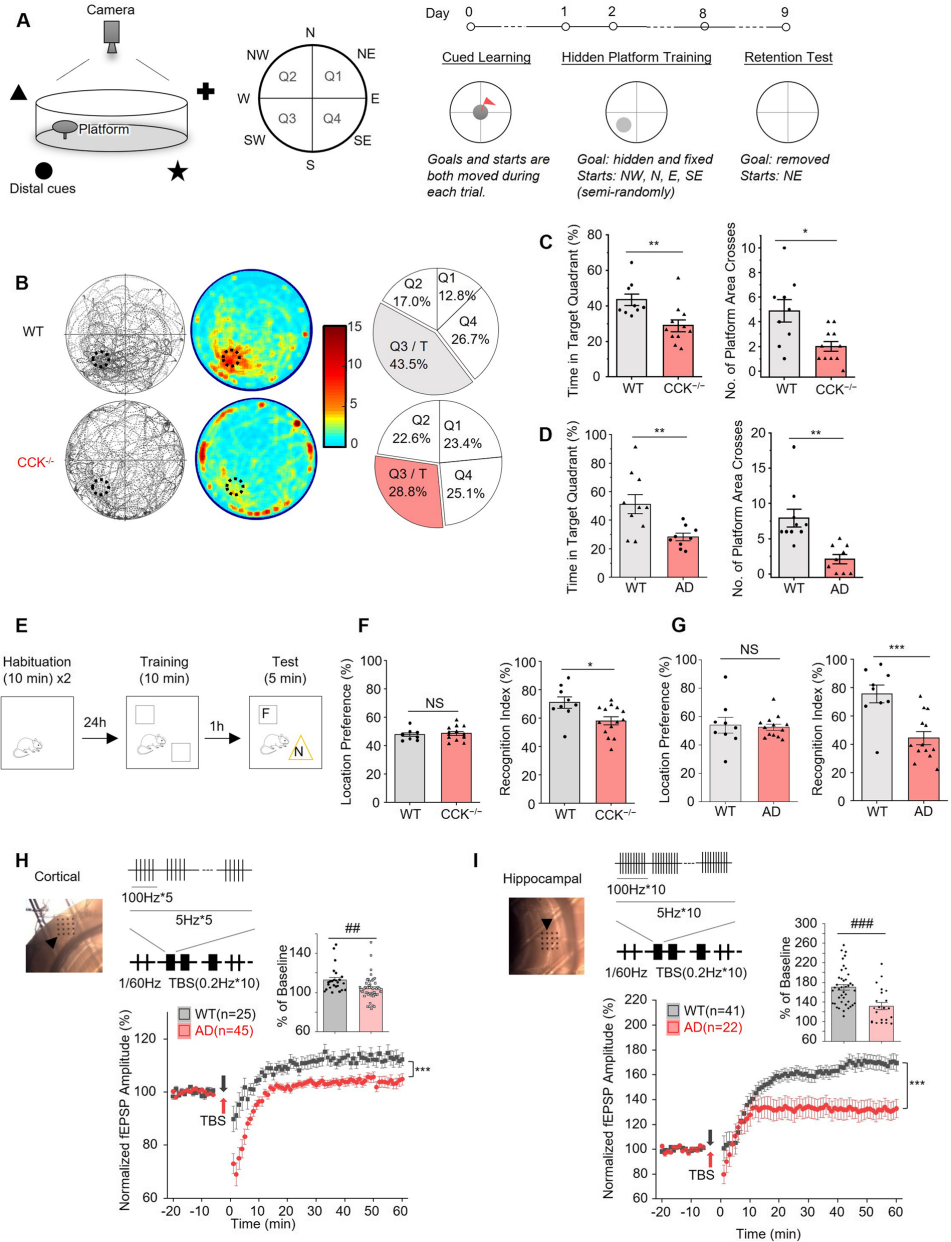
CCK^−/−^ and aged 3xTg AD mice exhibit comparable impaired cognition and neuroplasticity. **A** Schematic diagram of Morris Water Maze (MWM) and the experiment protocol. **B** Performance of WT (upper) and CCK^−/−^ mice (lower) in the MWM retention test. Left: Trajectory maps of mice's retrieval performance. The target platform area is indicated by the dotted circle. Middle: Heatmap visualization of spatial occupancy. Heatmaps were set to the same scale to facilitate comparison across groups. The redder colour represents increasing time spent. Right: Pie charts show the percentage of exploring time mice spent in each quadrant. Quadrant 3 (Q3) is the target quadrant (T), where the hidden platform was positioned during the training session. **C** CCK^−/−^ mice display a decreased percentage of time that mice spent in the target quadrant (left) and less times to cross the platform area (right) in the MWM retention test compared with WT mice. **D** Aged 3xTg AD mice developed impaired memory retrieval in the MWM retention test compared with aged WT mice. **E** Diagrammatic drawing and experimental protocol of the novel object recognition (NOR) test. F, familiar object; N, novel object. **F** CCK^−/−^ mice show no location preference in the NOR training (left) and a smaller recognition index in the NOR test (right) compared with WT mice. Location preference represents the percentage of time mice explored the left object in the training session. Recognition index represents the percentage of time mice explored the novel object in the test session. **G** Aged 3xTg AD mice show no location preference in the NOR training (left) and a smaller recognition index in the NOR test (right) compared with aged WT mice. **H** and (**I**) Scatter plots show the normalized amplitudes of fEPSP before and after TBS (indicated by arrows) on cortical (**H**) and hippocampal (**I**) slices of aged WT and aged 3xTg AD mice. A representative cortical slice showing the recording sites (black dots) and the stimulating site (indicate by arrow), and the TBS stimulation paradigm are shown above. The bar charts show the normalized amplitudes of fEPSPs for the last fifteen minutes of recordings. *p* value, NS > 0.05, * < 0.05, **, ## < 0.01, ***, ### < 0.001 by two-tailed two-sample t-test (**C**, **D**, **F**, **G**, and bar charts in **H** and **I**), or two-way repeated measures ANOVA with post-hoc Fisher test (scatter plots in **H** and **I**). Data are presented as the mean ± SEM. See also Figure S1 and S2

The MWM test was conducted as the protocol showed in Fig. 1c, which was adopted and revised based on Vorhees’s protocol [29]. Briefly, a submerged platform was placed in the middle of one of the quadrants of a round-shape swimming pool. Four conspicuous spatial cues were set surrounding the pool. During training days, every mouse received four training trials per day, with a 10 to 20-min gap between each trial. On the next day after training, animals will be released in the opposite quadrant, and their movements will be recorded. In the drug related experiments, the mouse was treated with drug candidates via intraperitoneally (i.p.) injection before training on each training day. The times of i.p. injection was depending on the half-life of drugs, for example, CCK-4, whose half-life is less than 5 min, was needed to be administrated four times before the four trials daily, while HT-267, whose half-life is more than 90 min, was needed only one administration to cover the four trials daily. Video recordings were analyzed with MATLAB software to analyze the animals’ movement and occupancy in each quadrant. Performance of all trials from the same group of animals were pooled together.

CCK-4 was purchased from Abcam (catalog no. ab141328). HT-267 was designed and provided by Guangzhou Institutes of Biomedicine and Health, Chinese Academy of Sciences. The drugs were dissolved in normal saline solution (0.9% NaCl) with less than 0.2% DMSO (catalog no. D8418, Sigma-Aldrich, German) to help dissolution. Vehicle (VEH) control was normal saline solution with the same concentration of DMSO as drug treatment group. Concentration and volume for i.p. injection were computed according to total blood volume (TBV). Mouse has about 56 µl of blood per grams of bodyweight [30]. For example, a mouse body weighted at 30 g, its TBV is 1.68 ml, and i.p. volume of 20 µM CCK-4 solution and 320 µM HT-267 solution for the mouse is 8.4 µl and 20 µl, respectively. Insulin syringes (BD 6 mm x 31G; 3/10 mL; catalog no. 324909) were used for i.p. injection.

### Novel object recognition (NOR) test

Rodents display a natural preference for novel objects. The apparatus was a box (25 cm × 25 cm × 25 cm) made of acrylic plates placed in a separate room. We captured and analyzed videos for animal behaviours. The experiment included three phases: habituation, training, and testing. During training, mice were allowed to explore two identical objects for 10 min. One hour later, one of the two familiar training objects was replaced with a novel object for testing. If the mouse recognizes the familiar object, it will spend most of its time on the novel object, which reflects its normal recognition memory. Recognition index to novel object was computed to evaluate the rescuing effect on recognition memory. Drugs were administrated into mice 2 min before the training trial via i.p. injection. The concentration and volume of the drugs are same as that in the MWM test.

### In vitro Electrophysiology

Mice was anaesthetized with gaseous isoflurane and decapitated to harvest their brains. The whole brain was obtained and immediately stored in ice-cold artificial cerebrospinal fluid (ACSF) (124 mM NaCl, 26 mM NaHCO_3_, 10 mM glucose, 3 mM KCl, 2 mM CaCl_2_, 1.25 mM KH_2_PO_4_ and 1.25 mM MgSO_4_, pH 7.4) (bubbled with 95%/5% O_2_/CO_2_) for brain sectioning. Brain slices containing cortical and hippocampal region were immediately transferred to an incubator filled with 95%/5% O_2_/CO_2_ bubbled ACSF at 28 ℃ for recovery of two hours and then applied to *in-vitro* recording. Three sets of commercial 4-slice 16-channel electrodes array systems (MED, Panasonic Alpha-Med Sciences) were used for high-throughput extracellular field potential recordings. One channel of microelectrodes was chosen as the stimulating electrode. The stimulation intensity during baseline (stable recording for at least 15 min) was adjusted to elicit 30%—50% of the maximal response. Theta-burst stimulation (TBS) was adopted to induce LTP at an intensity that can evoke 75%—90% of the maximal response. Afterwards, the stimulation intensity was adjusted back to baseline level to record for another hour. For drug treatment experiments, drug was dissolved in ACSF to 100 nM and administrated directly to the incubation solution. All multichannel electrophysiological data will be analyzed offline by the MED Mobius software.

### Real-time PCR

Tissues of these interested brain regions (EC, hippocampus, and neocortical areas) were dissected freshly from animals. The mRNAs were extracted from the tissues using TRIZOL (catalog no. 15596018, Invitrogen, Waltham, MA) and reversed transcribed into cDNA with Maxima Reverse Transcriptase (Thermo). CCK or CCKBR cDNA was then amplified specifically with QuantStudio™ 3 Real-Time PCR System (ABI). The sequences of the primers were: CCK-forward, 5’—AGC GCG ATA CAT CCA GCA G—3’; CCK-reverse, 5’—ACG ATG GGT ATT CGT AGT CCT C—3’; CCKBR-forward, 5’—ACC CTT TAT GCG GTG ATC TTT C—3’; CCKBR-reverse, 5’—GGT GAC CGT TCT TAG GCG TC—3’. The relative expressive level of the target genes was computed using the ΔΔCt method with control GAPDH. Two biological replicates were performed. Expressions of CCK mRNA levels was normalized to the average value of samples from the control group.

### Immunohistochemistry

Mice were deeply anesthetized with pentobarbital sodium (50 mg/kg, i.p.) and sequentially perfused 30 mL of 1 × phosphate-buffered saline (PBS) followed by 30 mL of 4% (w/v) paraformaldehyde (PFA). The brains were subsequently removed and post-fixed in 4% PFA at 4 °C overnight. After cryoprotection of the brains with 30% (w/v) sucrose, coronal sections (40 μm) were cut on a cryostat (Leica CM1860, Germany). Then the brain slices were rinsed three times with PBS and blocked with blocking buffer (10% goat serum in PBS with 0.2% Triton X-100) for 1.5 h at 37 ºC. Sections were incubated with anti-NeuN monoclonal antibody (Rabbit 1:2000, Abcam, ab177487, RRID: AB_2532109) at 4ºC for 48 h. After washing four times in PBS (each time for 10 min), sections were incubated with secondary antibody Alexa Fluor® 594 (Goat anti-Rabbit 1:500, Jackson ImmunoResearch Inc., 111–585-144, RRID: AB_2307325) for 1.5 h at 37ºC. Then the brain slices were rinsed with PBS three times before mounted on a glass slide with 70% glycerol in PBS. Fluorescent images were taken by a Nikon Eclipse Ni-E upright fluorescence microscope (4x, Nikon, Japan) and confocal microscope (20x, 40x, Nikon, Japan). As regards imaging analysis of quantification of neuron numbers, we used the Fiji software (https://imagej.net/Fiji).

### In vitro calcium imaging assay

All measurements of calcium imaging were performed on GPCRs overexpressed CHO cells by EnVision 2104 Multilabel Reader. Each well of 5 × 10^4^ cells that grown overnight on 96-well plates was measured by Fluo-8 No Wash Calcium Assay Kit (AAT-bioquest) to detect intracellular free Ca^2+^. Cells were cultured by DMEM/F-12 containing 10% FBS in 37 ℃ and 5% CO_2_ atmosphere and washed with DMEM/F-12 once after overnight incubation followed by incubating 30 min in 37 ℃ and 5% CO_2_ atmosphere with 100 µl Fluo-8 solution. To balance the cells at room temperature, they were incubated with Fluo-8 at room temperature for half an hour, followed by EnVision 2104 Multilabel Reader (Perkin Elmer) detection with an excitation wavelength of 485 nm and an emission wavelength of 535 nm. All curves and EC50 were fitted by GraphPad Prism.

### Pharmacokinetics study

Male wildtype mice weighing 25-30 g were utilized in the studies. The compound HT-267 was dissolved mixed in the solution containing 2% DMSO, 4% ethanol, 4% Cremophor EL and 90% ddH_2_O. Pharmacokinetic properties of animals were determined following intravenous administration. Animals were randomly distributed into 9 experimental groups (*n* = 4). The animals were dosed 5 mg/kg by intravenous injection. After single administration, whole blood samples (200 μl) and brain were obtained from the orbital venous plexus at the following time points after dosing: 2 min, 5 min, 10 min, 20 min, 40 min and 1 h, 2-h, 4-h, 6-h. Whole blood samples and brain were collected in heparinized tubes. The plasma fraction was immediately separated by centrifugation (8000 rpm, 6 min, and 4 °C) and stored at -20 °C until LC–MS-MS analysis. The mice were humanely euthanised with carbon dioxide without pain. For the standard curve, HT-267 was dissolved in DMSO at a concentration of 2 mg/ml and diluted with 100% solution (methanol:H_2_O 1:1) to series concentration. 10 μl series concentration solution and 50 μl blank plasma were added to 1.5 ml tube and vortexed for 3 min, then 150 μl acetonitrile containing internal standard were added and vortexed for 5 min, finally spin tube in centrifuge at 13000 g for 40 min at 4 °C. The final concentrations were as follow: 5, 10, 20, 50, 100, 200, 500, 1000, 2000, 5000 ng/ml. The plasma and brain samples were prepared using protein precipitation method. 10 μl acetonitrile solution and 50 μl plasma samples or brain samples were added to 1.5 ml tube and vortexed for 3 min, then 150 μl acetonitrile containing internal standard were added and vortexed for 5 min, finally spin tube in centrifuge at 6000 g for 40 min at 4 °C. After centrifuge, 100 μl supernatant was transfer to the 96 well plates and analyzed by LC–MS/MS using Agilent 1290 Infinity II high performance liquid chromatography, Agilent Technologies 6470Triple Quad LC/MS.

### Statistical analysis

Data shown is mean ± SEM. All statistical analysis was done in Origin 2018 (OriginLab, USA) and SPSS (IBM, USA). Statistical significance was set at *p < 0.05, **p < 0.01 and ***p < 0.001. Specific assessment, one-way ANOVA, two-way repeated measures ANOVA, two tailed two-sample student’s t-test was illustrated in the corresponding sections. Statistical figures (bar charts, pie charts, box plots and scatter charts) were plotted by Origin 2018 (OriginLab, USA) and GraphPad Prism. Trajectory maps and heatmaps were generated by MATLAB.

## Results

### CCK^−/−^ and aged 3xTg AD mice exhibit comparable impaired cognition and neuroplasticity

The current study adopted two mouse models as the subjects: male CCK^−/−^, C57/BL/6 as its wildtype control, and male aged 3xTg (12 months old), 129S1/SvImJ at the same age as its wildtype control.

We first examined the spatial learning and memory retention using MWM tests (Paradigm in Fig. 1A) of the CCK^−/−^ and aged 3xTg mice compared with their wildtype control. Both groups showed no significant differences in the escape latency in the cued learning task with a visible platform in different quadrants compared with their wildtype control (D0 in the paradigm of Fig. 1A, data in Figure S1A and S2A).

The animals underwent the 8-day hidden-platform training. The CCK^−/−^ mice showed a significantly slower learning curve in finding the hidden platform than their wildtype control (Figure S1B, *p* < 0.05, between groups, two-way repeated measures ANOVA; Day 8, *p* < 0.05, post hoc Fisher test). This learning deficit is also confirmed in aged 3xTg mice (Figure S2B, *p* < 0.01, between groups, two-way repeated measures ANOVA; Day 5 and 6, *p* < 0.05, Day 7 and 8, *p* < 0.01, post hoc Fisher test). On day 9, the CCK^−/−^ mice demonstrated an apparent deficit in the memory retention test after removing the hidden platform (Fig. 1B and C). The superimposed swimming traces or the heatmap show that the CCK^−/−^ mice had almost no memory about the hidden platform, while the wildtype control (WT in the figure) remembered (Fig. 1B), and the CCK^−/−^ group spent 28.8% of the time, at the chance level in the target quadrant, while wildtype 43.5% of the time (Fig. 1C; time spent in the target quadrant, CCK^−/−^ 28.8% vs WT 43.5%, *p* < 0.01; numbers of crossing the platform area, CCK^−/−^ 2.0 vs WT 4.89, *p* < 0.05, two-sample t-test). The impaired memory retrieval is also confirmed in aged 3xTg mice (Fig. 1D; time spent in the target quadrant, AD 28.3% vs WT 51.2%, *p* < 0.01, two-sample t-test; the number of times that animals crossed the platform area, AD 2.56 vs WT 7.50, *p* < 0.01, two-sample t-test).

The second behavioural experiment we adopted to examine the cognitive function was the NOR test based on the rodents’ natural tendency to explore novel objects (Fig. 1E). The exploration time that animals spent on each object was analyzed, and there was no difference in the proportion of time spent on the two objects during training between the CCK^−/−^ and the wildtype control groups (Fig. 1F, CCK^−/−^ 48.6% vs WT 47.8%, *p* > 0.05, two-sample t-test). However, a significant difference was observed in the proportion of time spent on the novel object during the test session (Fig. 1F, CCK^−/−^ 57.9% vs WT 71.0%, *p* < 0.05, two-sample t-test), indicating a deficit of the CCK^−/−^ mice in remembering the familiar object that was presented earlier. Similarly, aged 3xTg mice displayed impaired recognition memory in the NOR test (Fig. 1G, WT 75.6% vs AD 44.3%, *p* < 0.001, two-sample t-test) without difference of the location preference in the training (Fig. 1G, WT 53.8% vs AD 52.4%, *p* > 0.05, two-sample t-test) compared with the aged wildtype mice.

Next, we used the multielectrode array system to examine the neuroplasticity of neocortical and hippocampal slices of AD and wildtype brains. We found a significantly lower potentiation in the AD cortical slices than the control group at the end of the 60-min recording after theta-burst stimulation (TBS)-induced LTP protocol (Fig. 1H; TBS protocol in the inset; WT 112.8% vs AD 104.1%, *p* < 0.0001, two-way repeated measures ANOVA, post-hoc Fisher test). The hippocampal slice of the AD mice also showed a significantly lower potentiation in the TBS-induced LTP than its wildtype control (Fig. 1I, WT 169.6% vs AD 131.7%, *p* < 0.0001, two-way repeated measures ANOVA, post-hoc Fisher test). Previously we found that high-frequency stimulation cannot induce in vivo neocortical LTP in the CCK^−/−^ mice [24]. Further in this study, we discovered that TBS cannot induce in vitro cortical and hippocampal LTP in the CCK^−/−^ mice compared with wildtype mice (Figure S1D-E).

In conclusion, the CCK^−/−^ and aged 3xTg AD mice exhibited comparable behavioural impairments in the MWM and NOR tests and physiological deficits in the neuroplasticity assay.

### Administration of CCK-4 alleviated the deficits of CCK^−/−^ mice

**Fig. 2:**
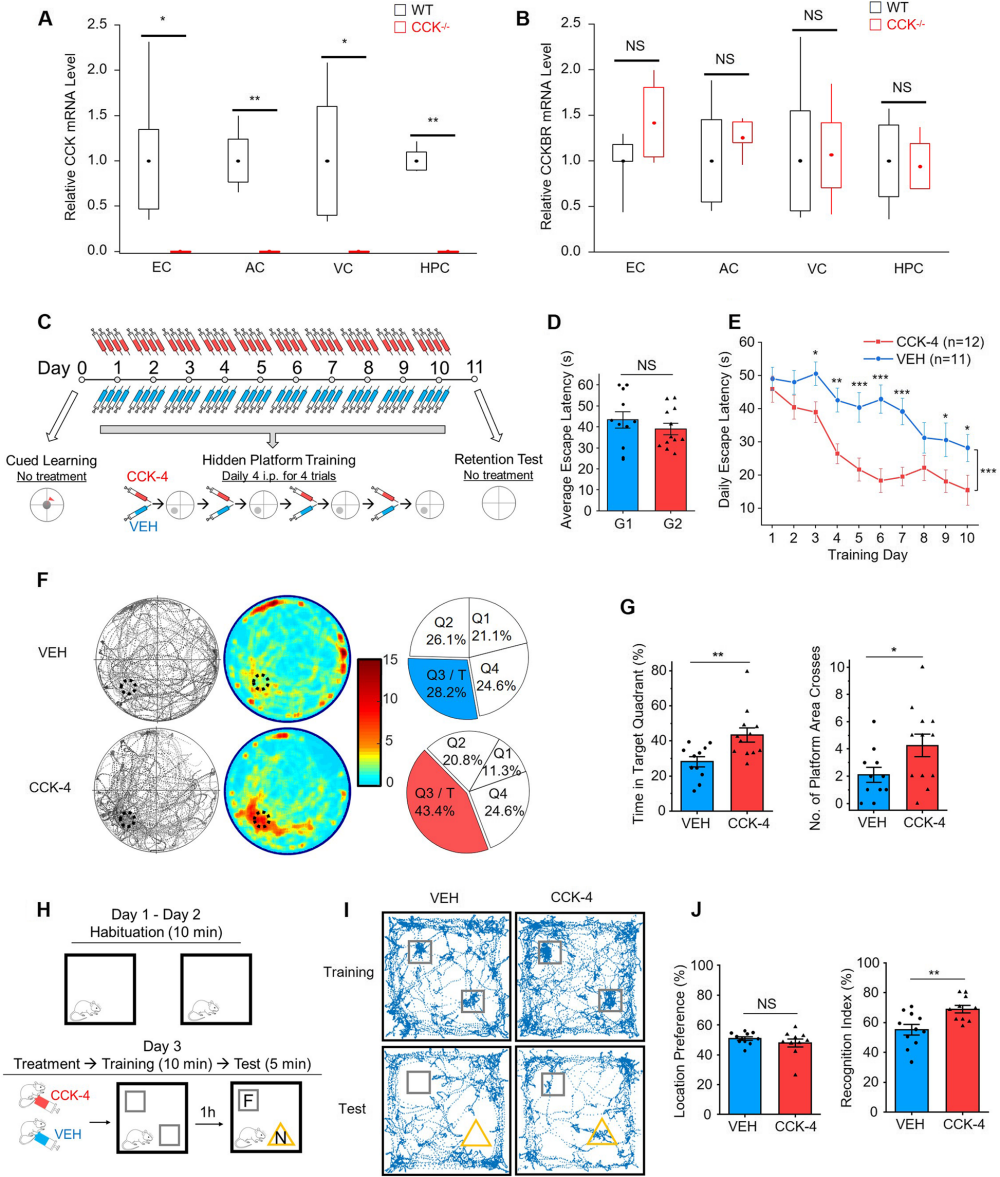
Administration of CCK-4 alleviated the deficits of CCK^−/−^ mice. **A** and **B** The relative quantity of CCK (**A**) and CCKBR (**B**) mRNA levels in different brain regions of CCK^−/−^ and WT control mice. Expressions of the mRNA levels was normalized to the average value of samples from the corresponding WT group of each brain region. EC, entorhinal cortex, AC, auditory cortex, VC, visual cortex, HPC, hippocampus. Box charts show 25 and 75 percentiles with boxes, 10 and 90 percentiles with whiskers, and means with dots. **C** Schematic diagram of Morris Water Maze (MWM) test. In the hidden platform training, CCK-4 or vehicle (VEH) was given intraperitoneally (i.p.) every time before each trial, and four doses were given to each mouse on each training day. **D** Average escape latency in MWM cued learning of two randomly divided groups (G1 and G2) of CCK^−/−^ mice. G1 and G2 were assigned to receive VEH or HT-267 treatments, respectively, in the following training session. The results were averaged across the five trials for each mouse. **E** Acquisition performance of CCK-4 or VEH treated CCK^−/−^ mice in the MWM hidden platform training process. The results were averaged across four trials for each mouse and averaged across mice per day. **F** Trajectory maps (left), heatmaps (middle) and pie charts (right) show mice’s performance in the MWM retention test of CCK-4 (lower) or VEH (upper) treated CCK^−/−^ mice. **G** The percentage of time mice spent in the target quadrant (left) and the number of platform area crosses (right) of CCK-4 or VEH treated CCK^−/−^ mice in the MWM retention test. **H** Diagrammatic drawing and experimental protocol of the novel object recognition (NOR) test. F, familiar object; N, novel object. One dose of CCK-4 or VEH was given by i.p. injection to each mouse before training. **I** Examples of trajectories of the VEH or CCK-4 treated CCK^−/−^ mice in the NOR training and test sessions. Squares indicate the positions of two identical objects in the training and the familiar object in the test. Triangles indicate the positions of the novel object in the test. **J** Location preference (left) in the NOR training session and recognition index (right) in the test session of the VEH or CCK-4 treated CCK.^−/−^ mice. p value, NS > 0.05, * < 0.05, ** < 0.01, *** < 0.001 by two-tailed two-sample t-test (**A**, **B**, **D**, **G** and **J**), or two-way repeated measures ANOVA with post-hoc Fisher test (**E**). Data are presented as the mean ± SEM. See also Figure S3

The CCK^−/−^ mice had zero mRNA of CCK but comparable mRNA level of CCKBR (Fig. 2A-B). We hypothesized that the CCK agonist administration to the CCK^−/−^mice during the training session rescues the learning and remembering impairments and hence improves their performance in the retention test of the MWM and NOR tests. We adopted the following CCKBR agonist, CCK-4, with a half-life of 13 min in the human and < 1 min in the rat plasm in the rescuing experiments [31]. It is reported that CCK-4 can penetrate the blood–brain barrier after i.p. injection [26]. With the short half-life of CCK-4 in the rodent plasm, we injected CCK-4 or VEH control before each of the 4-trial training for all 10 training days in the MWM test, with the hope that CCKBRs were activated for all trials (Fig. 2C).

CCK^−/−^ mice were assigned randomly to two groups and there is no difference in average escape latency in the MWM cued learning between two groups (Fig. 2D). In the hidden platform training, the experimental group with CCK-4 injection showed significantly improved learning compared with the VEH group (Fig. 2E, p < 0.001, between groups, two-way repeated measures ANOVA; Days 4, 9 and 10, *p* < 0.05, Day 4, *p* < 0.01, Day 5, 6 and 7, *p* < 0.001, post hoc Fisher test). The CCK-4 injected group demonstrated a noticeable improvement in the memory retention test after removing the hidden platform (Fig. 2F) and a significantly more exploring time in the target quadrant than the VEH-injected group (Fig. 2G; time spent in the target quadrant, CCK-4 treated group 43.4% vs VEH treated group 28.2%, *p* < 0.01; numbers of crossing the platform area, CCK-4 treated group 4.3 vs VEH treated group 2.1, *p* < 0.05; two-sample t-test).

We also examined the rescuing effect of CCK-4 in the NOR test (Fig. 2H). We injected CCK-4 or VEH control into CCK^−/−^ mice of the two groups, respectively. There was no difference in the proportion of time spent on the two objects between the CCK-4 injected and the VEH-injected groups during the training session (Fig. 2J, CCK-4 treated group 47.8% vs VEH treated group 51.0%, *p* > 0.05, two-sample t-test). However, a significant difference of the recognition index between the two groups was observed in the test session (Fig. 2J, CCK-4 treated group 69.0% vs VEH treated group 55.3%, *p* < 0.01, two-sample t-test). The CCK-4-injected mice spent more time near the novel object, indicating that they remembered the familiar object that was presented earlier, and CCK-4 rescued the learning deficit of the CCK^−/−^ mice.

Besides, we found that infusion of CCK-4 rescued the deficits in LTP induction (Figure S3, from 109.1% to 144.5% in cortical slices, *p* < 0.001, and from 117.0% to 160.1% in hippocampal slices at the end of the 60-min recording, *p* < 0.001, two-way repeated measures ANOVA, post-hoc Fisher test).

In sum, CCK-4 successfully rescued the deficits of learning, memory, and neuroplasticity of the CCK^−/−^ mice.

### Application of CCK-4 rescued impaired cognition and neuroplasticity of aged 3xTg AD mice

**Fig. 3:**
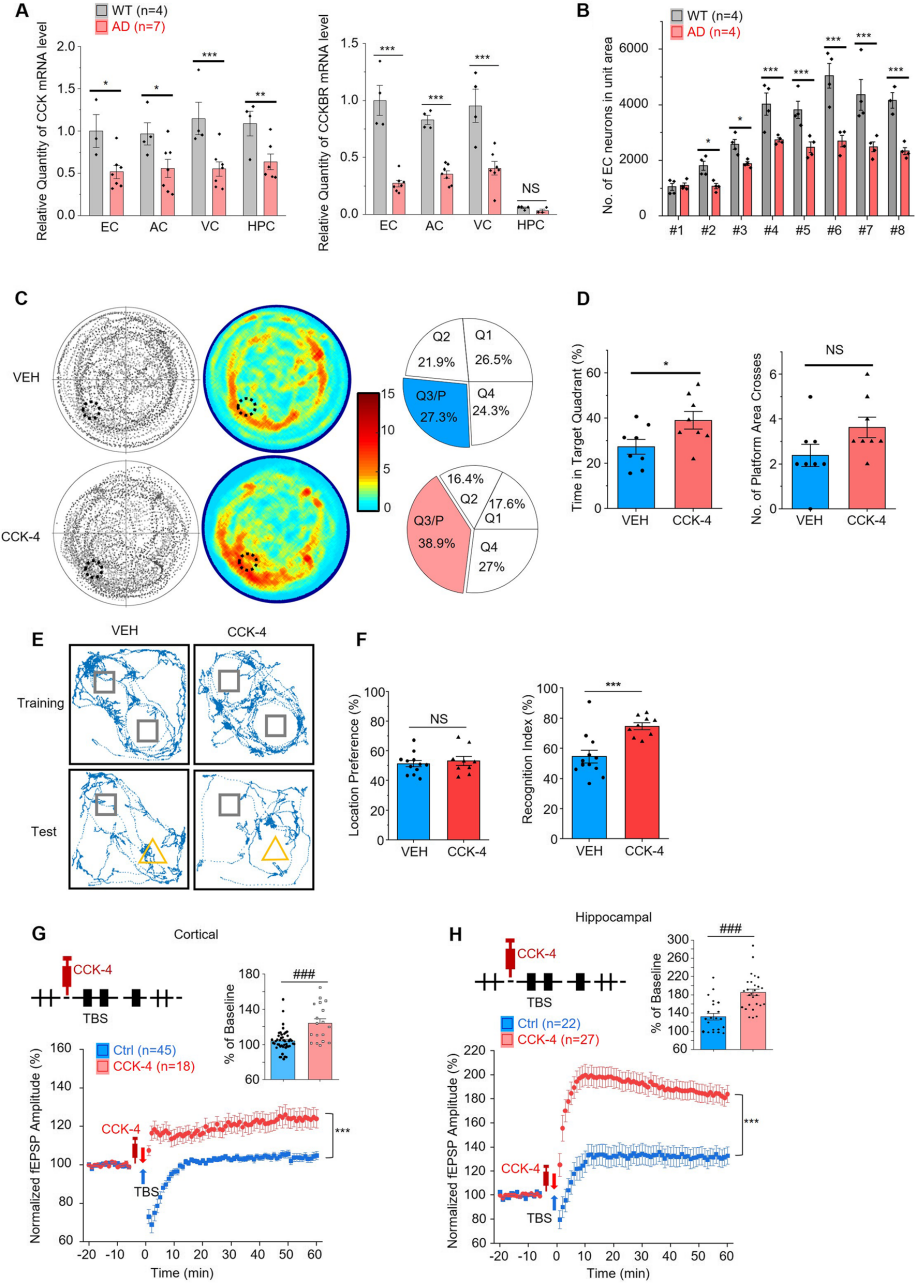
Application of CCK-4 rescued impaired cognition and neuroplasticity of aged 3xTg AD mice. **A** The relative quantity of CCK (left) and CCKBR (right) mRNA levels in different brain regions of aged 3xTg AD (AD) and control wildtype (WT) mice. Data were normalized to the average of EC samples of WT mice. EC, entorhinal cortex, AC, auditory cortex, VC, visual cortex, HPC, hippocampus. **B** The number of EC neurons in the unit area of the eight positions of the brain slices. The eight positions are: #1 -2.18 mm posterior to bregma (Bre -2.18), #2 Bre -2.46, #3 Bre -2.80, #4 Bre -3.08, #5 Bre -3.40, #6 Bre -3.80, #7 Bre -4.16, and #8 Bre -4.48. **C** Trajectory maps (left), heatmaps (middle) and pie charts (right) show mice’s performance in the MWM retention test of CCK-4 (lower) or VEH (upper) treated 3xTg mice. **D** The percentage of time mice spent in the target quadrant (left) and the number of platform area crosses (right) of CCK-4 or VEH treated 3xTg mice in the MWM retention test. **E** Examples of trajectories of the VEH or CCK-4 treated 3xTg mice in the NOR training and test sessions. Squares indicate the positions of two identical objects in the training and the familiar object in the test. Triangles indicate the positions of the novel object in the test. **F** Location preference (left) in the NOR training session and recognition index (right) in the test session of the VEH or CCK-4 treated 3xTg mice. **G** and **H** Scatter plots show the normalized amplitudes of fEPSP before and after TBS on cortical (**G**) and hippocampal (**H**) slices with or without CCK-4 application of aged 3xTg mice. The bar charts show the normalized amplitudes of fEPSPs for the last fifteen minutes of recordings. p value, NS > 0.05, * < 0.05, ** < 0.01, ***, ### < 0.001 by two-tailed two-sample t-test (**D**, and bar charts in **G** and **H**), or two-way repeated measures ANOVA with post-hoc Fisher test (**A**, **B**, and scatter plots in **G** and **H**). Data are presented as the mean ± SEM. See also Figure S4 and S5

We examined the mRNA expression in the EC and other brain regions of aged 3xTg AD mice compared to the wildtype control. We found that the mRNA levels of both CCK and CCKBR in the EC, as well as in the neocortex of 3xTg AD mice were significantly downregulated compared to wildtype control (Fig. 3A). However, only the mRNA level of CCK, rather than CCKBR, in the hippocampus of 3xTg AD mice was significantly less than the wildtype control (Fig. 3A). Moreover, we examined the number of neurons in the EC, and we discovered that the whole number and the number in the unit area of EC neurons in aged 3xTg AD mice were both significantly less than in WT mice (Fig. 3B and S4A).

In the MWM test, identical to the above experiment on CCK^−/−^ mice, we injected CCK-4 or VEH control to aged 3xTg mice before each of the 4-trials during all 10 training days in the MWM test, hoping that CCKBRs were activated for all trials (Fig. 2A). The experimental group with CCK-4 injection showed some improvement in learning compared to the VEH group (Figure S5B). The CCK-4 injected group demonstrated a marginal improvement but was statistically significant in the memory retention test after removing the hidden platform (Fig. 3C-D). CCK-4-injected 3xTg mice spent significantly more time in the target quadrant than the VEH-injected group (Fig. 3D; time spent in the target quadrat, CCK-4 treated group 38.9% vs VEH treated group 27.3%, *p* < 0.05, two-sample t-test; numbers of crossing the platform area, CCK-4 treated group 3.6 vs VEH treated group 2.4, *p* > 0.05, two-sample t-test).

We also examined the rescuing effect of CCK-4 on aged 3xTg mice in the NOR test (Fig. 3E-F). Two groups of 3xTg mice received CCK-4 or VEH injection, respectively, during the training session and no difference of location preference was detected between the two groups (Fig. 3F, CCK-4 treated group 53.1% vs VEH treated group 51.3%, *p* > 0.05, two-sample t-test). However, CCK-4 treated mice displayed a significantly higher recognition index in test session than the VEH-treated mice (Fig. 3F, CCK-4 treated group 74.7% vs VEH treated group 54.6%, *p* < 0.001, two-sample t-test), indicating that CCK-4 rescued the learning deficit of aged 3xTg mice.

Moreover, we found that infusion of CCK-4 rescued the deficits in LTP induction of the aged 3xTg mice (Fig. 3G-H, from 104.1% to 124.3% in cortical slices, *p* < 0.001, and from 131.7% to 184.6% in hippocampal slices at the end of the 60-min recording, *p* < 0.001, two-way repeated measures ANOVA, post-hoc Fisher test).

Taken together, these results proved that CCK-4 successfully rescued the learning, memory, and neuroplasticity deficits of aged 3xTg mice.

### HT-267, an analogue of CCK-4 with a longer half-life, showed great potency to CCKBR

**Fig. 4:**
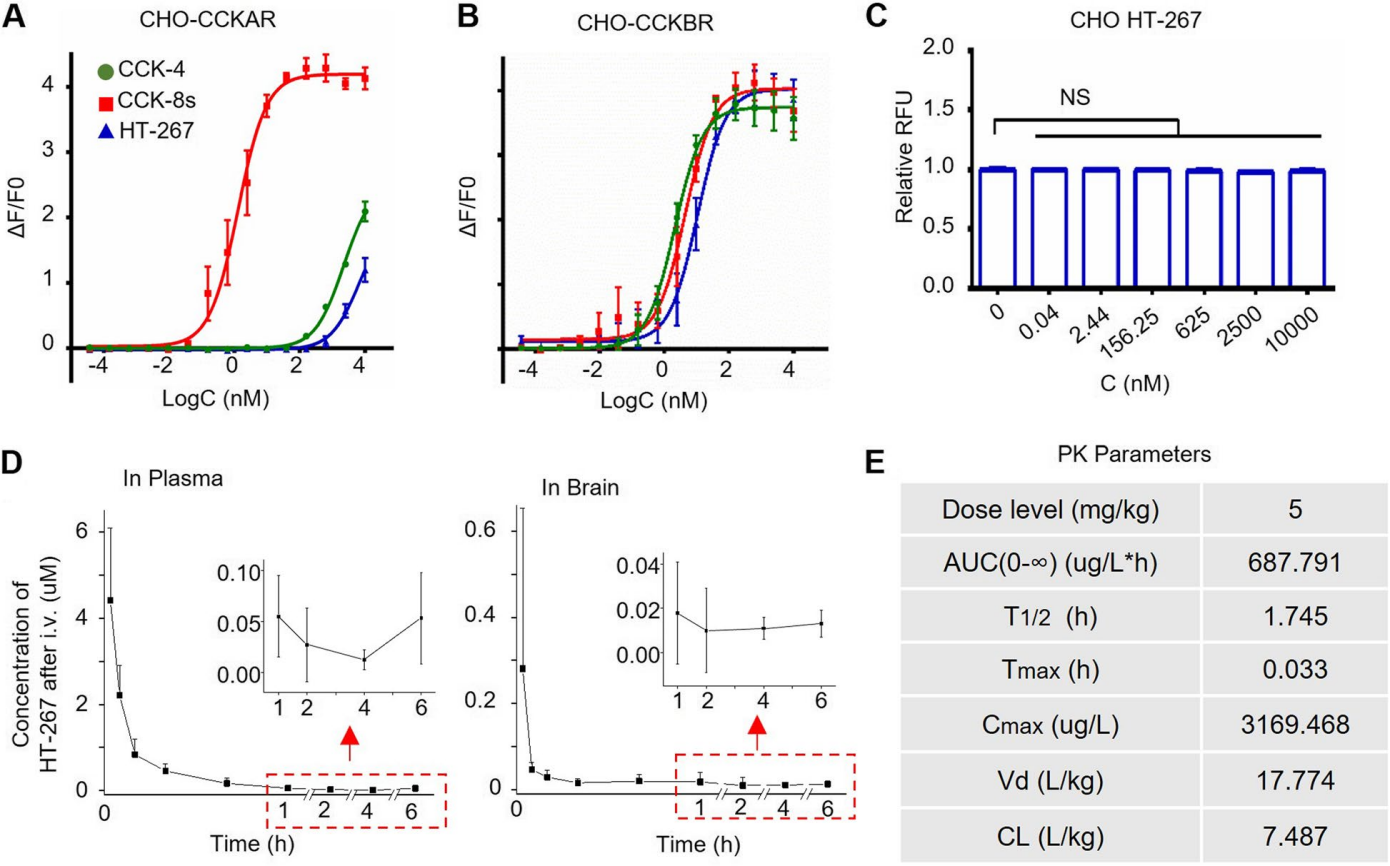
HT-267, an analogue of CCK-4 with a longer half-life, showed great potency to CCK BR. **A** EC50 curve of CCK4, CCK8s and HT-267 for the CHO-CCKAR cells. EC50 (CCK4) = 2267.5 ± 492.694 nM, EC50 (CCK8s) = 1.741 ± 1.115 nM, EC50 (HT-267) = 8171.25 ± 2319.157 nM. 100% CCK8s maximal response of CCK4 = 48.058 ± 3.417%, 100% CCK8s maximal response of HT-267 = 27.518 ± 4.176%. **B** EC50 curve of CCK4, CCK8s and HT-267 for the CHO-CCKBR cells. EC50 (CCK4) = 2.182 ± 0.623 nM, EC50 (CCK8s) = 5.079 ± 2.423 nM, EC50 (HT-267) = 11.552 ± 4.534 nM. 100% CCK8s maximal response of CCK4 = 91.302 ± 6.370%, 100% CCK8s maximal response of HT-267 = 96.750 ± 6.282%. **C** The response of different concentrations of HT-267 was measured by calcium imaging assay in CHO cells. Relative RFU (Relative RFU = RFUt = 30 s / RFUt = 0: the RFU ratio of the 30 s after (RFUt = 30 s) and before (RFUt = 0) adding HT-267/HHBS). ∆F/F0 ((RFUt = 30 s-RFUt = 0)/RFUt = 0). **D** Pharmacokinetics (PK) results of the concentration–time curve of HT-267 in plasma and brain of male KM mice following intravenous (i.v.) administration. **E** The parameters of HT-267 in the PK study. *p* value, NS > 0.05 by one-way ANOVA with post-hoc Fisher test (**C**). Data are presented as the mean ± SEM

Conceptually we demonstrated the therapeutical effect of CCK-4 for deficient cognition in CCK^−/−^and aged 3xTg mice. However, CCK-4 is far from being a drug candidate due to its short half-life in the plasm of the rodents. In the earlier experiments, we had to inject it for 4 times during the daily training of 4-trial hidden-platform searching. To improve the stability of the peptide CCK-4, we modified its chemical structure and named it HT-267 [32]. It contains two natural amino acids and two unnatural amino acids. Specifically, the N-terminal acetylation and C-terminal amidation were also applied and thus increased the stability of HT-267. We found that the analogue, HT-267, worked as a CCKBR agonist with high potency at the nM level when we used a CHO CCKBR cell line (Fig. 4A-C). HT-267 had an agonistic activity with the EC50 of 11.552 ± 4.534 nM to CCKBR (Fig. 4B).

Our pharmacokinetics studies show that HT-267 lasts a very long period after intravenous administration (Fig. 4D), which also indicated that HT-267 penetrated the blood–brain barrier. HT-267 had a half-life of 1.745 h (Fig. 4E), much longer than the CCK-4 in the mouse plasm. With longer half-life and great potency to CCKBR, HT-267 possesses great potential as the drug candidate to replace CCK-4.

### HT-267 rescued the deficiencies of learning, memory, and neuroplasticity of aged AD mice

**Fig. 5:**
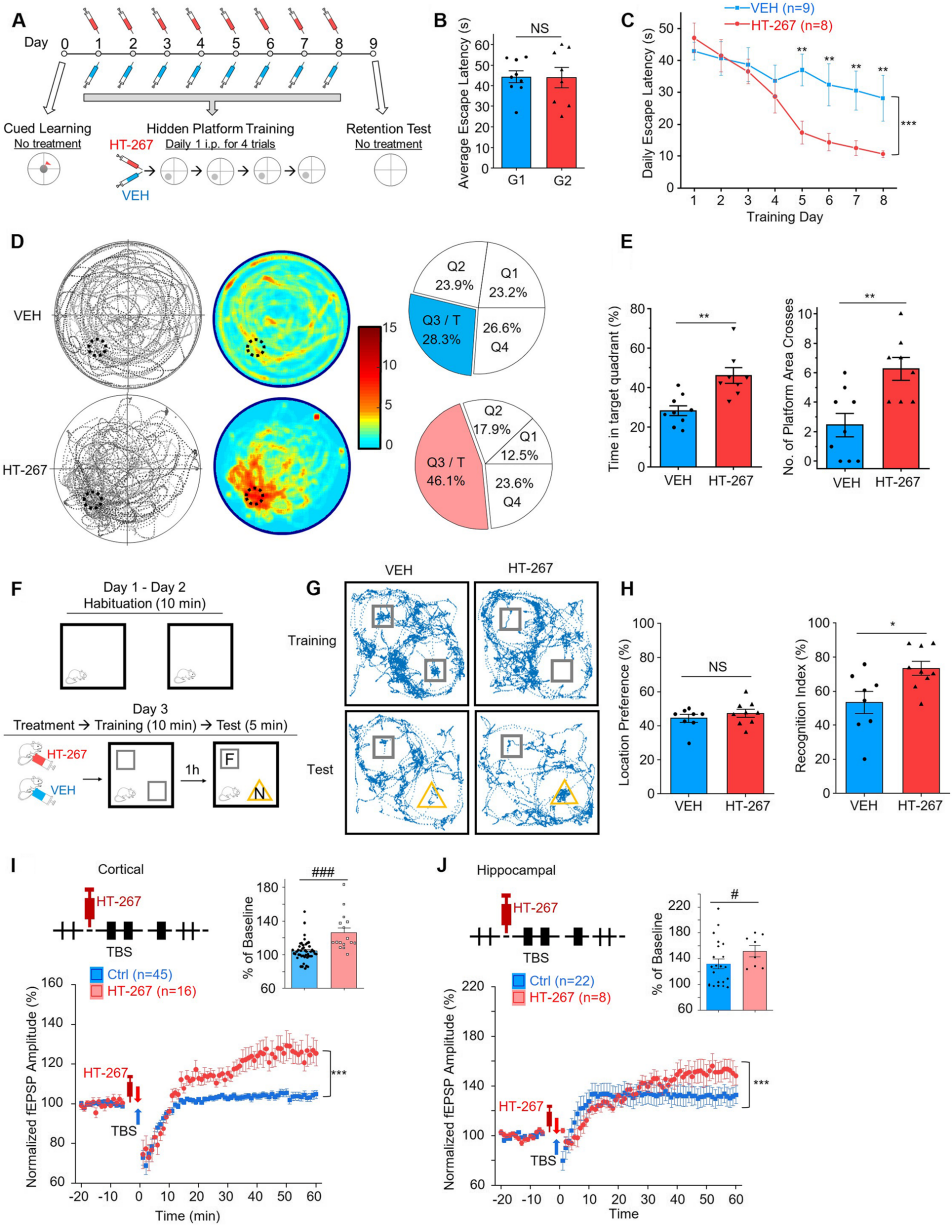
HT-267 rescued the deficiencies of learning, memory, and neuroplasticity of aged AD mice. **A** Schematic diagram of Morris Water Maze (MWM). One dose of HT-267 or VEH (VEH) was given intraperitoneally (i.p.) to each mouse before training on each training day. **B** Average escape latency in MWM cued learning of two randomly divided groups (G1 and G2) of 3xTg mice. G1 and G2 were assigned to receive VEH or HT-267 treatments, respectively, in the following training session. **C** Acquisition performance of HT-267 or VEH treated 3xTg mice in the MWM hidden platform training. **D** Trajectory maps (left), heatmaps (middle) and pie charts (right) show mice’s performance in the MWM retention test of HT-267 (lower) or VEH (upper) treated 3xTg mice. **E** The percentage of time mice spent in the target quadrant (left) and the number of platform area crosses (right) of HT-267 or VEH treated 3xTg mice in the MWM retention test. **F** Diagrammatic drawing and experimental protocol of the novel object recognition (NOR) test. F, familiar object; N, novel object. One dose of HT-267 or VEH was given by i.p. injection to each mouse before training. **G** Examples of trajectories of the VEH or HT-267 treated 3xTg mice in the NOR training and test sessions. Squares indicate the positions of two identical objects in the training and the familiar object in the test. Triangles indicate the positions of the novel object in the test. **H** Location preference (left) in the NOR training session and recognition index (right) in the test session of the VEH or HT-267 treated 3xTg mice. **I** and **J** Scatter plots show the normalized amplitudes of fEPSP before and after TBS on cortical (**I**) and hippocampal (**J**) slices with or without HT-267 application of aged 3xTg mice. The bar charts show the normalized amplitudes of fEPSP for the last fifteen minutes of recordings. *p* value, NS > 0.05, *, # < 0.05, ** < 0.01, ***, ### < 0.001 by two-tailed two-sample t-test (**B**, **E**, **H**, and bar charts in **I** and **J**), or two-way repeated measures ANOVA with post-hoc Fisher test (**C**, and scatter plots in **I** and **J**). Data are presented as the mean ± SEM. See also Figure S6

First, we investigated the effect of HT-267 to aged 3xTg mice on the deficiencies of learning and memory. Aged 3xTg mice were randomly divided into two groups, and the two groups of mice showed no difference in MWM cued learning (Fig. 5B). As HT-267 had a much longer half-life in the mouse plasm, we modified our protocol for the frequency of drug application (Fig. 5A), in which we injected HT-267 and its VEH control once a day. Considering the prolonged half-life and high binding affinity to CCKBR of HT-267, one dose of treatment could cover the four training trials for the mouse. The experimental group with HT-267 injection showed a significant improvement in the learning process compared with the VEH group (Fig. 5C, *p* < 0.001, between groups, two-way repeated measures ANOVA; pairwise comparison of HT-267 and the VEH-treated mice on Day 5, Day 6, Day 7 and Day 10, **p < 0.01, post-hoc Fisher test). The HT-267-injected group demonstrated a significantly improved memory retention test after the hidden platform was removed (Fig. 5D-E). The HT-267-injected group spent considerably more time in the target quadrant than the VEH-injected group (Fig. 5E; time spent in the target quadrat, HT-267 treated group 46.1% vs VEH treated group 28.3%, *p* < 0.01; the number of crossing the platform area, HT-267 treated group 6.1 vs VEH treated group 2.6, *p* < 0.01, two-sample t-test). In the NOR test (Fig. 5F), there was no difference of the location preference between the HT-267-injected and the VEH-injected groups during the training session (Fig. 5H, HT-267 treated group 47.2% vs VEH treated group 44.3%, *p* > 0.05, two-sample t-test) but a significant difference of the recognition index during the test session (Fig. 5H, HT-267 treated group 73.4% vs VEH treated group 53.6%, *p* < 0.05, two-sample t-test). Next, we found that infusion of HT-267 rescued the deficits in LTP induction (Fig. 5I-J, from 104.1% to 126.1% in cortical slices, *p* < 0.001, and from 131.7% to 151.7% in hippocampal slices at the end of the 60-min recording, *p* < 0.001, two-way repeated measures ANOVA, post-hoc Fisher test). These results demonstrated that the treatment of HT-267 rescued the impaired cognition and neuroplasticity of aged 3xTg mice.

We summarized the performance on the two behavioural experiments of the aged wildtype, aged 3xTg and aged 3xTg with CCK-4 or HT-267 application. The HT-267 treated aged 3xTg mice showed similar performance to the wildtype control mice in the MWM test (Figure S6). Both CCK-4 and HT-267 treated 3xTg mice showed significantly improved performance in the MWM and NOR tests (Figure S6A-B). We also summarized the performance on the cortical and hippocampal LTP inducements of the aged wildtype, aged 3xTg, aged 3xTg with CCK-4 or HT-267 application, CCK^−/−^, and CCK^−/−^ with CCK-4 application (Figure S6C-D). CCK^−/−^ and aged 3xTg mice exhibit lower LTP compared to WT mice, and the applications of both CCK-4 and HT-267 enhanced the cortical and hippocampal LTP. In summary, CCKBR agonist, CCK-4 and HT-267 as a novel drug, successfully rescued the deficits of learning, memory, and neuroplasticity of CCK^−/−^ and aged 3xTg mice.

## Discussion

The present study demonstrated that CCK^−/−^ and aged 3xTg AD mice develop impairments not only behaviourally in the MWM and NOR tests but also physiologically in the neuroplasticity assay. Intraperitoneal CCK-4 injection successfully rescued the deficits of CCK^−/−^ mice and aged 3xTg mice. To improve the stability of CCK-4, we synthesized its analogue, HT-267, which worked as a CCKBR agonist with high potency and longer half-life. HT-267 presents great rescue effects on the deficiencies of learning, memory, and neuroplasticity of aged 3xTg AD mice.

The 3xTg AD mouse model has been widely used in basic pathological studies and new anti-AD drug development. Their memory deficit characteristics have been illuminated in previous works [33, 34]. Once again, we investigated their deficits in learning and memory, and additionally, deficiency in neuroplasticity with *in-vitro*electrophysiology. Although we used only male 3xTg AD mice, it is reported that 3xTg mice aged 12 months old develop enough Aβ pathology to qualify for AD study as proposed previously [33, 35, 36].

The first symptom of AD patients with cognitive impairments is the decline of recent memory and progression to anterograde amnesia [14, 37]. Anterograde amnesia is categorized as difficulty in forming new long-term memory. Both MWM and NOR tests are suited to screening anterograde amnesia [29, 38].

Plagman and colleagues examined cerebrospinal fluid CCK levels in 287 AD subjects and discovered that higher CCK was related to better memory scores and a decreased likelihood of AD impairment [28]. Our earlier studies found that the entorhinal cortex enables sound-sound and visuoauditory associative memories in the auditory cortex via the released CCK from their neocortical projection terminals [24, 25]. It is reasonable to speculate that the reduced CCK level causes deficiency in recent memory or anterograde amnesia. Cerebral atrophy in the EC is the first change in early AD patients [39–41]. In the present study, we found that CCK mRNA level in the EC of aged 3xTg mice is reduced compared with their wildtype control. CCK^−/−^ mice exhibited impairments in learning and memory similarly to the aged 3xTg AD mice. The similarity of their behavioural performance between the CCK deficient and aged 3xTg mice further strengthens the link between the CCK decline and memory deficiency.

The mRNA expression of CCKBR in CCK^−/−^ mice is comparable to that of their wildtype control. Administrating CCK-4 rescued the performance of learning and memory in both MWM and NOR tests. Though CCKBRs are downregulated in the aged 3xTg mice, the rescuing effect of CCK-4 is similar to that of CCK^−/−^ mice. These results indicate that the deficiencies of learning and memory of aged 3xTg mice are likely associated with the declined CCK production in the EC due to the brain atrophy of the region [39–41]. These results also indicate that the 12-month-old 3xTg mice have a relatively intact signal pathway in the downstream, as learning and memory can be rescued by the addition of CCKBR agonists. We may conclude that the deficiencies of 12-month-old 3xTg mice are mainly caused by signal pathways before the CCK release.

Our earlier studies have also demonstrated that CCK is the key to HFS-induced LTP in the neocortex [17, 24]. It is reasonable to associate the learning deficiency with the LTP deficiency. Our results in Fig. 1 strongly indicate the link between the deficiencies in LTP and the performances of MWM and NOR tests of aged 3xTg mice. Again, the rescuing effect of CCK agonists in LTP induction demonstrates that the downstream pathway for learning and neuroplasticity of the aged 3xTg mice is not devastatingly affected. The rescued LTP reached about 140% in the neocortex and nearly 180% in the hippocampus, which is on par with young wildtype mice, indicating the downstream pathway is likely intact.

Available AD drugs, including donepezil, galantamine, rivastigmine and memantine, designed to target these pathological structures, including the amyloid plaque and tau tangle in the brain tissue, can only alleviate the symptoms of AD patients at an early stage [42, 43]. Although Aβ targeting antibodies such as aducanumab claim they can interfere with the pathological progress of AD, they were approved after decades of development [44]. The proposed CCKBR agonist has a new strategy for rescuing the neuroplasticity at the downstream pathway of memory formation. The drug candidate HT-267 targeted specifically anterograde amnesia, one of the major symptoms of AD. Although the physiological etiology of CCK in AD still needs to be further investigated, this study sheds light on a potential pharmaceutical candidate for AD and dementia. We hope that our candidate will exert similar therapeutic effects on AD patients as in the transgenic mouse model, thus compliment the currently available AD drugs. Greatly decreased neural activity is observed in the AD patients' brains compared with their healthy controls [1–3]. Since HT-267 targets rescuing the symptom of anterograde amnesia of AD patients with brain atrophy, especially of the medial temporal lobe, it is expected, though to be examined, to promote the general activity of the neocortex and thus retard the secondary brain atrophy spreading into it.

## Conclusions

Here we provide more evidence to support the role of CCK in learning and memory. We discovered that aged 3xTg AD mice exhibited reduced CCK mRNA expression in the entorhinal cortex but reduced CCKBR expression in the neocortex and hippocampus. We demonstrated that aged 3xTg AD and CCK knock-out mice have impairments cognitively in two behavioural tests and physiologically in the neuroplasticity assay, and importantly, they displayed improved performance and enhanced long-term potentiation after the treatment of CCK-4. Moreover, we elaborated on the rescue effect of a promising novel drug, HT-267, on impaired synaptic and cognitive functions of aged 3xTg AD mice. This study sheds light on a potential pharmaceutical candidate for AD and dementia. The physiological etiology of cholecystokinin in AD can be studied to further clarify the correlation between them.

### Supplementary Information

Supplementary Material 1.

Supplementary Material 2.

Supplementary Material 3.

## Data Availability

The datasets used and/or analyzed during the current study are available from the corresponding author on reasonable request.

## Abbreviations

AD: Alzheimer’s disease
CCK: Cholecystokinin
CCKBR: Cholecystokinin B receptor
CCK^−/−^: CCK knock-out
EC: Entorhinal cortex
HFS: High-frequency stimulation
LTP: Long-term potentiation
MWM: Morris water maze
NOR: Novel object recognition
CCK-4: CCK tetrapeptide
i.p.: Intraperitoneally
TBV: Total blood volume
ACSF: Artificial cerebrospinal fluid
TBS: Theta-burst stimulation
PBS: Phosphate-buffered saline
PFA: Paraformaldehyde
WT: Wildtype
ANOVA: Analysis of variance
VEH: Vehicle
