# Supplementary material for: Cholecystokinin B receptor agonists alleviates anterograde amnesia in cholecystokinin-deficient and aged Alzheimer's disease mice
Source: Alzheimers Res Ther. 2024 May 15;16:109. doi: 10.1186/s13195-024-01472-1 (PMC11094875; doi:10.1186/s13195-024-01472-1)
Supplement: Supplementary file 1 — Supplementary Material 1. [file 13195_2024_1472_MOESM1_ESM.docx]

## Supplementary data


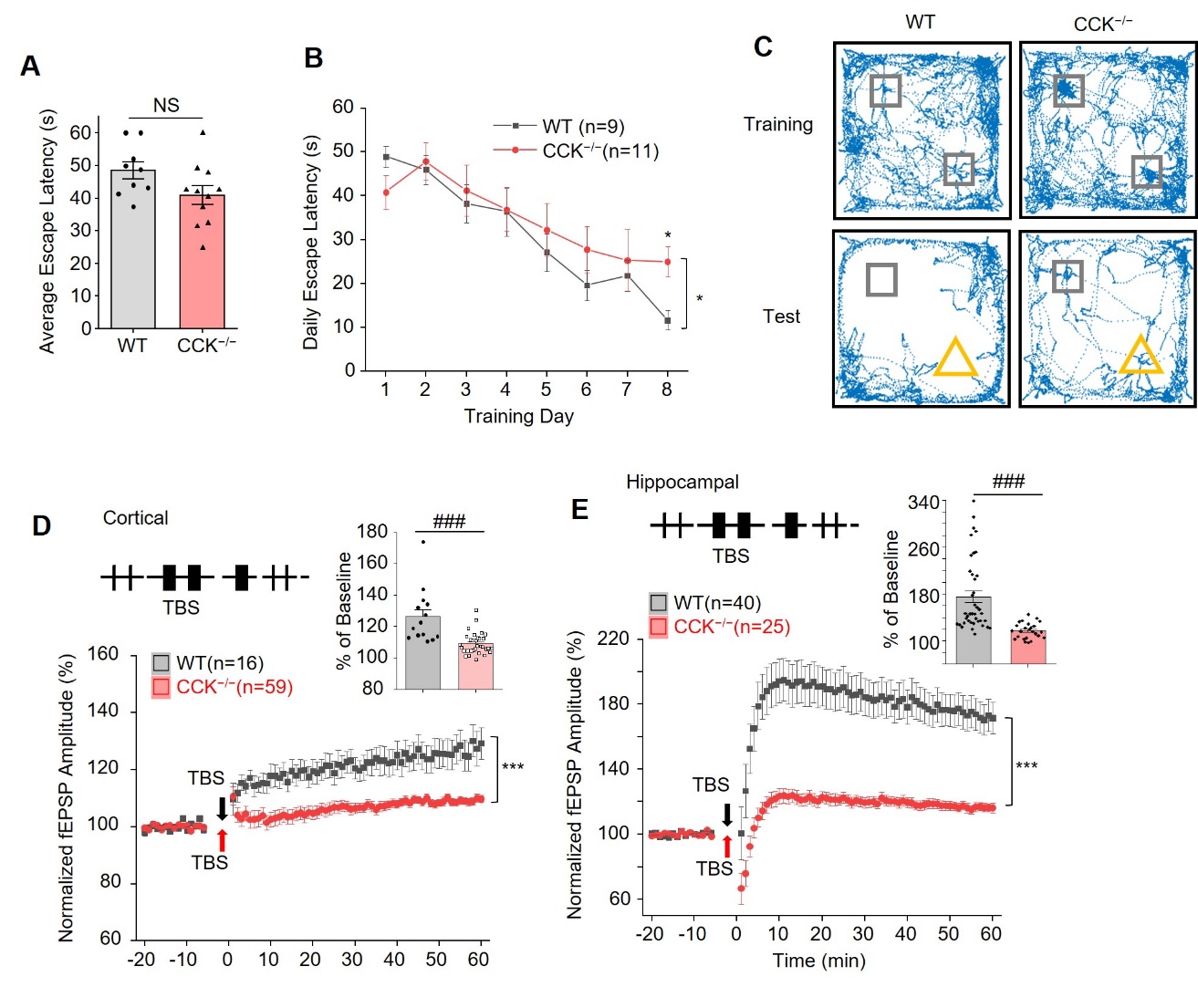


**Figure S1.** **CCK^−/−^** **mice displayed impaired cognition and neuroplasticity, related to Figure 1.**

(A) Average escape latency in MWM cued learning of CCK^−/−^ and control wildtype (WT) mice.

(B) Acquisition performance of aged 3xTg and control WT mice in the MWM hidden platform training process.

(C) Examples of trajectories of the aged 3xTg and control WT mice in the NOR training and test sessions. Squares indicate the positions of two identical objects in the training and the familiar object in the test. Triangles indicate the positions of the novel object in the test.

(D) and (E) Scatter plots show the normalized amplitudes of fEPSP before and after TBS on cortical (D) and hippocampal (E) slices of WT and CCK^−/−^ mice. The bar charts show the normalized amplitudes of fEPSP for the last fifteen minutes of recordings.

p value, NS > 0.05, * < 0.05, ** <0.01, ***, ### < 0.001 by two-tailed two-sample t-test (A, and bar charts in D and E), or two-way repeated measures ANOVA with post-hoc Fisher test (B, and scatter plots in D and E). Data are presented as the mean ± SEM.


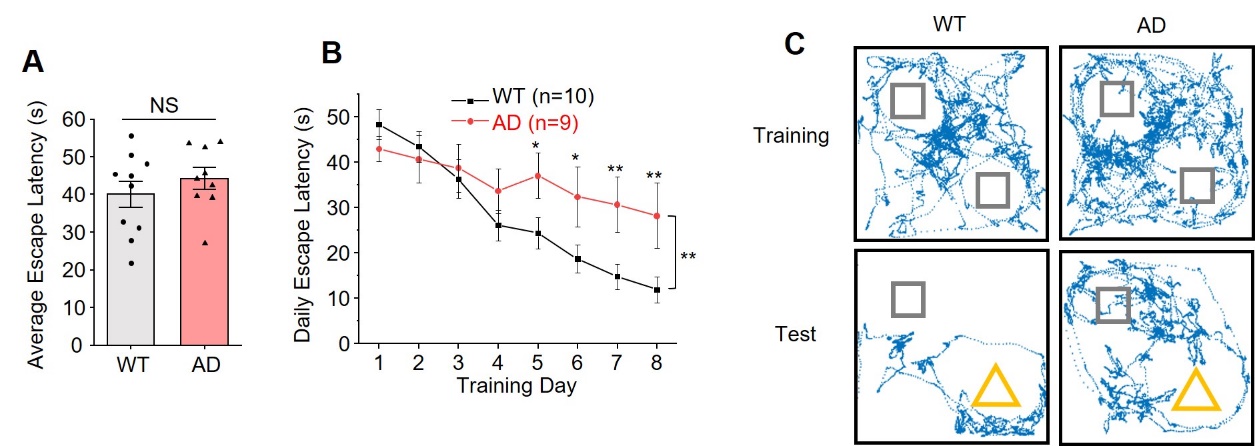


**Figure S2.** **Aged 3xTg AD mice displayed impaired learning in Morris water maze and novel object recognition tests, related to Figure 1.**

(A) Average escape latency in MWM cued learning of aged 3xTg AD and control wildtype (WT) mice.

(B) Acquisition performance of aged 3xTg and control WT mice in the MWM hidden platform training process. The results were averaged across four trials for each mouse and averaged across mice per day.

(C) Examples of trajectories of the aged 3xTg and control WT mice in the NOR training and test sessions.

p value, NS > 0.05, * < 0.05, ** <0.01 by two-tailed two-sample t-test (A), or two-way repeated measures ANOVA with post-hoc Fisher test (B). Data are presented as the mean ± SEM.


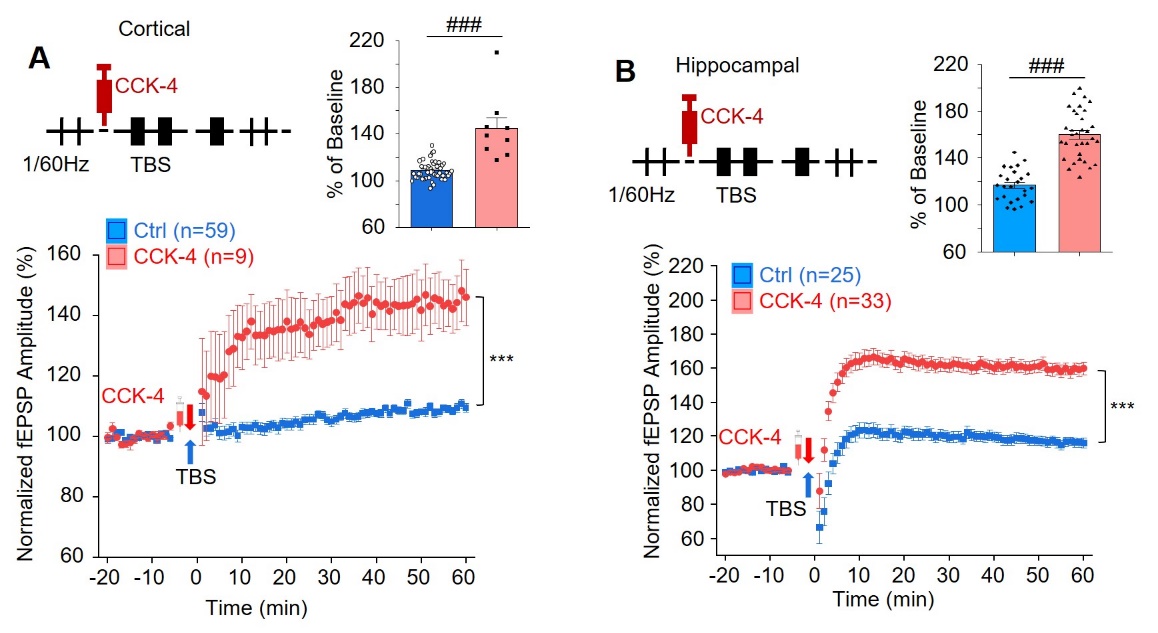


**Figure S3. CCK-4 applications restore cortical and hippocampal LTP of CCK^−/−^** **mice, related to Figure 2.**

(A) and (B) Scatter plots show the normalized amplitudes of fEPSP before and after TBS on cortical (A) and hippocampal (B) slices with or without CCK-4 application. The bar charts show the normalized amplitudes of fEPSP for the last fifteen minutes of recordings.

p value, ***, ### < 0.001 by two-tailed two-sample t-test (bar charts in A and B), or two-way repeated measures ANOVA with post-hoc Fisher test (scatter plots in A and B). Data are presented as the mean ± SEM.


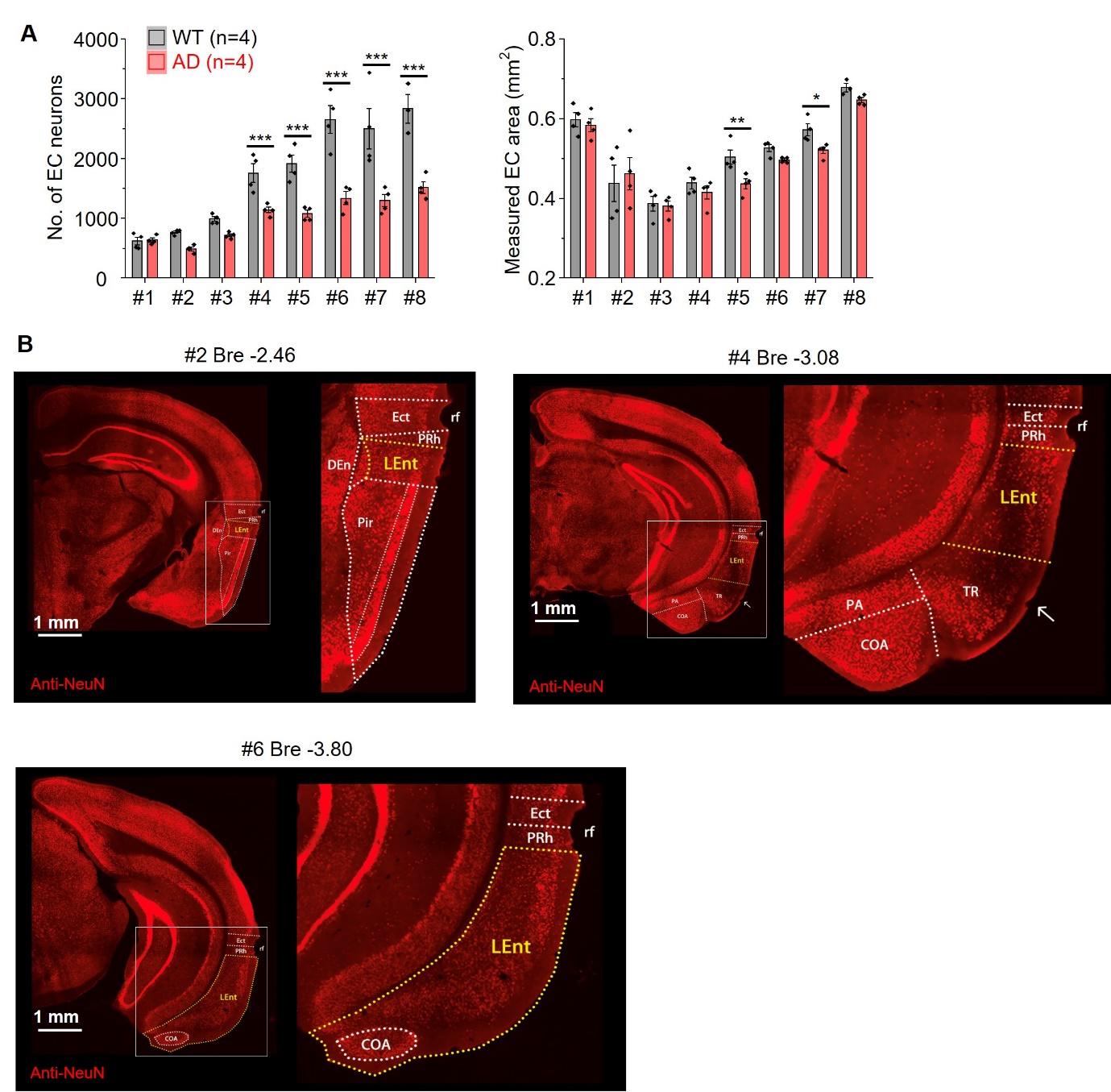


**Figure S4. The number of EC neurons were decreased in aged 3xTg AD mice compared with aged WT mice, related to Figure 3.**

(A) The number of EC neurons (left) and the EC area (right) of the eight positions of the brain slices. The eight positions are: #1 -2.18 mm posterior to bregma (Bre -2.18), #2 Bre -2.46, #3 Bre -2.80, #4 Bre -3.08, #5 Bre -3.40, #6 Bre -3.80, #7 Bre -4.16, and #8 Bre -4.48.

(B) Examples of the brain slice of aged 3xTg AD mice stained by Anti-NeuN antibody. The area circled by the yellow dotted line was applied for neuron counting. LEnt, lateral entorhinal cortex.

p value, * < 0.05, ** <0.01, *** < 0.001 by two-way repeated measures ANOVA with post-hoc Fisher test (A). Data are presented as the mean ± SEM.


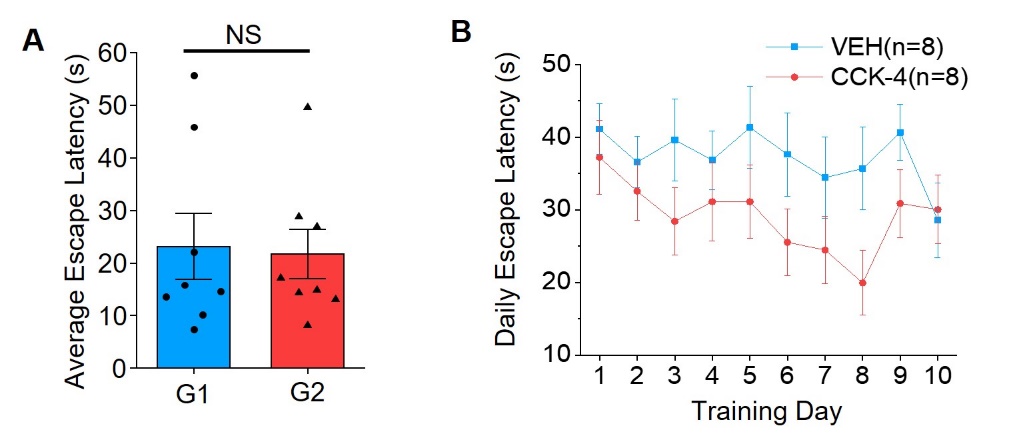


**Figure S5.** **Mice’s performance in cued learning and hidden platform training of MWM test of aged 3xTg AD mice, related to Figure 3.**

(A) Average escape latency in MWM cued learning of two randomly divided groups (G1 and G2) of 3xTg mice. G1 and G2 were assigned to receive VEH or CCK-4 treatments, respectively, in the following training session.

(B) Acquisition performance of CCK-4 or VEH treated 3xTg mice in the MWM hidden platform training.

p value, NS > 0.05 by two-tailed two-sample t-test (A). Data are presented as the mean ± SEM.


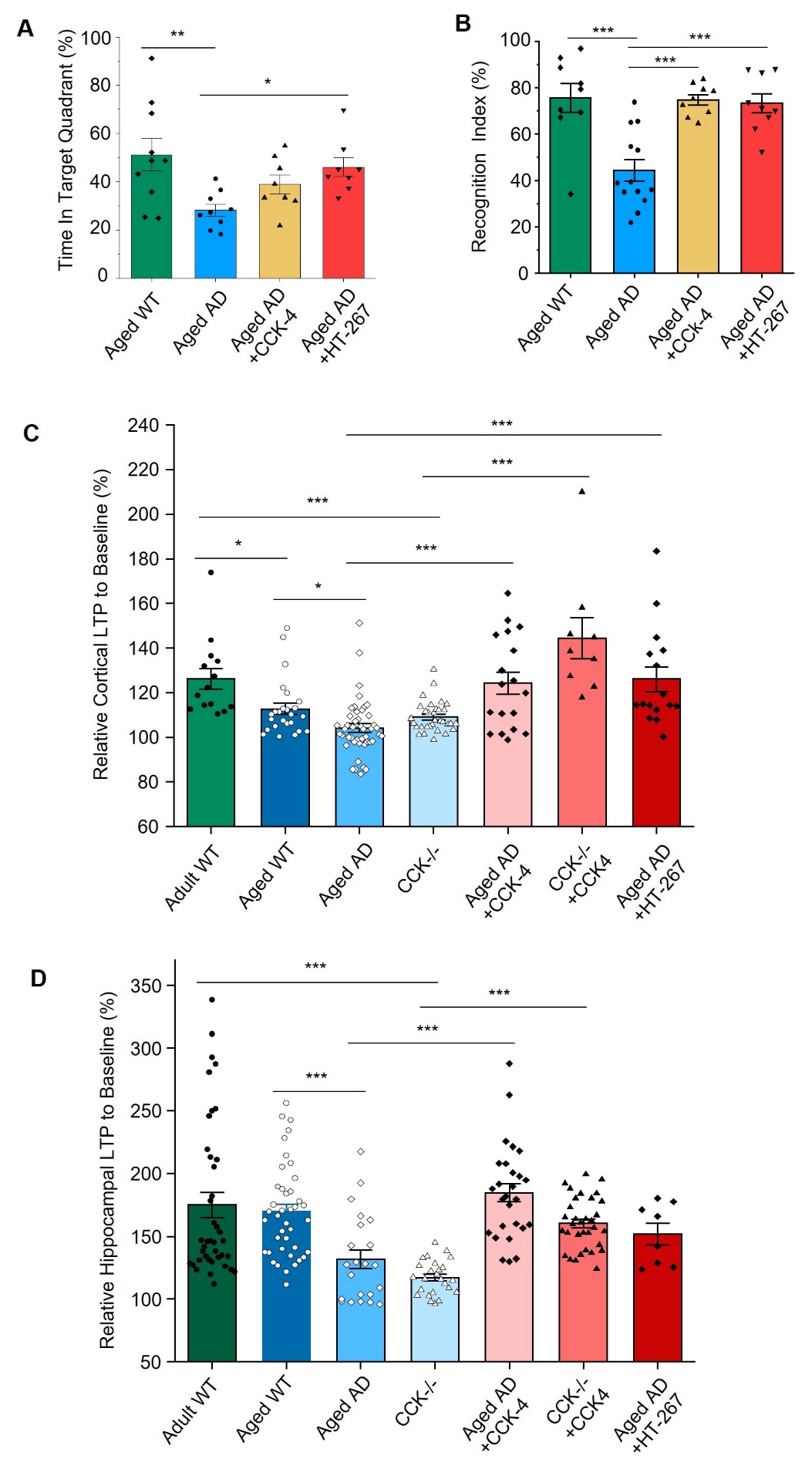


**Figure S6.** **Comparison of the drug effects on aged 3xTg AD and CCK^−/−^** **mice, related to Figure 5.**

(A) Percentage of time mice spent in target quadrant of MWM retention tests.

(B) Recognition index to a novel object in NOR testing sessions.

(C) and (D) Average of the last 15 minutes of the relative cortical (C) and hippocampal (D) LTP to the baseline.

p value, * < 0.05, ** <0.01, *** < 0.001 by one-way ANOVA with post-hoc Fisher test (A, B, C and D). Data are presented as the mean ± SEM.
